# When do we need to suspect maturity onset diabetes of the young in patients with type 2 diabetes mellitus?

**DOI:** 10.20945/2359-3997000000431

**Published:** 2022-01-14

**Authors:** Özlem Üstay, Esra Arslan Ateş, Tugçe Apaydin, Onur Elbasan, Hamza Polat, Gizem Günhan, Ceyda Dinçer, Lamia Şeker, Ayşegül Yabacı, Ahmet Ilter Güney, Dilek Gogas Yavuz

**Affiliations:** 1 Marmara University School of Medicine, Endocrinology and Metabolism Istanbul Turkey Marmara University School of Medicine, Endocrinology and Metabolism, Istanbul, Turkey; 2 Marmara University School of Medicine Istanbul Turkey Marmara University School of Medicine, Medical Genetics, Istanbul, Turkey; 3 Marmara University School of Medicine Istanbul Turkey Marmara University School of Medicine, Internal Medicine, Istanbul, Turkey; 4 Bezmialem Vakif University School of Medicine Department of Biostatistics Istanbul Turkey Bezmialem Vakif University School of Medicine, Department of Biostatistics, Istanbul, Turkey

**Keywords:** MODY, maturity onset diabetes of the young, early onset type 2 diabetes

## Abstract

**Objetivo:**

Maturity onset diabetes of the young (MODY) patients have clinical heterogeneity as shown by many studies. Thus, often it is misdiagnosed to type 1 or type 2 diabetes(T2DM). The aim of this study is to evaluate MODY mutations in adult T2DM patients suspicious in terms of MODY, and to show clinical and laboratory differences between these two situations.

**Subjects and methods:**

In this study, we analyzed 72 type 2 diabetic patients and their relatives (35F/37M) who had been suspected for MODY and referred to genetic department for mutation analysis. The gene mutations for MODY have been assessed in the laboratory of Marmara University genetics. Totally 67 (32F/35M; median age 36.1) diabetic patients were analyzed for 7 MODY mutations. Twelve patients who have uncertain mutation (VUS) were excluded from study for further evaluation. MODY(+) (n:30) patients and T2DM patients (n:25) were compared for clinical and laboratory parameters.

**Results:**

In MODY(+) subjects, mutations in GCK (MODY 2) (n:12; 40%) were the most common followed by HNF4A (MODY 1) (n:4; 13.3%). Diabetes diagnosis age was younger in MODY(+) group but not statistically significant. Sixty-six percent of MODY(+) subjects had diabetes history at 3-consecutive generations in their family compared with 28% of T2DM patients statistically significant (p:0.006). Gender, BMI, C-peptide, HbA1c, lipid parameters, creatinine, GFR, microalbuminuria, vitamin D and calcium were not statistically different between the groups.

**Conclusion:**

According to present study results, MODY mutation positivity is most probable in young autoantibody (-) diabetic patients diagnosed before 30 years of age, who have first degree family history of diabetes.

## INTRODUCTION

Type 2 diabetes mellitus (T2DM) incidence has been increasing in Turkey, as well as globally, and has begun to be seen at much earlier ages. Some of these patients have a genetic disposition for diabetes, just like their relatives. Maturity onset diabetes of the young (MODY) is an autosomal dominant inherited, common monogenic form of diabetes ( 1 , 2 ). Incidence of MODY varies by region ranging from 2% to 5% of all diabetic patients ( 1 , 3 ). MODY is caused by over 800 mutations in 14 different MODY-related genes ( 4 - 8 ). The most common mutations are found in *GCK* (glucokinase), *HNF1A* (hepatocyte nuclear factor-1alpha) and *HNF4A* (hepatocyte nuclear factor-4alpha) ( 2 , 9 ).

Many studies have shown the clinical heterogeneity of MODY patients ( 10 - 13 ). Thus, MODY is often misdiagnosed as type 1 or type 2 diabetes. The clinical diagnosis of MODY is based on young onset (before the age of 25), presence of diabetes in at least 3 consecutive generations, absence of β-cell autoantibodies, and relatively preserved endogenous insulin secretion, according to some studies ( 14 , 15 ). Since MODY is diagnosed at an early age, it is often confused with early-onset T2DM. Early-onset T2DM is recognized as a special kind of type 2 diabetes which was diagnosed at a young age (30-45 years) with various genetic tendencies. Although type 2 diabetes is a disease related to aging, the prevalence of early-onset T2DM in adults has increased globally ( 16 ). Due to overlapping clinical features, distinguishing MODY from early-onset T2DM is often difficult. The molecular diagnosis and classification of MODY patients are essential for a correct treatment decision and in the judgment of the prognosis. Genetic testing is highly specific and sensitive; therefore, it represents the gold standard for diagnosing MODY. However, genetic testing is an expensive procedure which renders it an inaccessible tool for diagnosing MODY. Thus, careful consideration is required when determining which patients should be tested; and considerable efforts have been made to investigate nongenetic or clinical markers in order to facilitate the differential diagnosis of MODY.

Studies have shown that *GCK* mutations are common in Turkish pediatric cohorts, which represent one-quarter of MODY cases of all diabetics in childhood ( 17 ). The pediatric population usually has a higher risk of type 1 diabetes mellitus (T1DM), and their clinical presentation is quite different in young patients from that of T2DM patients. Thus, it is relatively easy to differentiate these two diseases in pediatric groups. However, insufficient number of studies have been conducted related to the clinical differences between MODY and early-onset T2DM in adult populations. Moreover, contradictory data about the most common MODY mutation types appears in recent literature. *GCK* and *HNF1A* mutations are known to be the most common mutations, especially in Europe and North America ( 2 , 9 , 18 ). According to the limited data available in Turkey, *HNF1A* is regarded as the most common mutation in MODY patients, however there is insufficient information about the clinical characteristics of early-onset T2DM patients and differences from MODY ( 19 ).

The aim of this study was to evaluate MODY mutations in adult T2DM patients who were suspected of MODY, and to show clinical and laboratory differences between these two types of diabetes.

## SUBJECTS AND METHODS

In this study, we analyzed 72 T2DM patients and their relatives (35 F/37 M), followed at the endocrinology out-patient clinic of Marmara University Hospital, who had been suspected of MODY and referred to the genetic department for mutation analysis. Although the clinical diagnosis of MODY is usually based-on young onset before the age of 25, presence of diabetes in at least 3 consecutive generations, absence of β-cell autoantibodies (anti-GAD antibody, anti-islet cell antibody), and relatively preserved endogenous insulin secretion, the specific clinical criteria for MODY diagnosis are still not very clear. In our study, we enrolled all patients referred to the genetic department for MODY mutation, thus not all the patients met all the clinical criteria for MODY diagnosis described in the literature.

The study was approved by the local ethics committee of Marmara University School of Medicine (protocol number 09.2020.01).

All patients were invited to the endocrinology outpatient clinic and were evaluated for age, duration of diabetes, age at diagnosis, micro and macro complications of diabetes, family history of diabetes, drug usage (type and exposure time), body mass index (BMI), and blood pressure; and were tested for fasting plasma glucose, fasting serum C-peptide level, glycosylated hemoglobin (HbA1c), lipid profile (total, LDL-cholesterol, HDL-cholesterol and triglycerides), islet cell autoantibodies (ICAs), glutamic acid decarboxylase (GAD) autoantibodies and insulin antibodies (IAAs).

Positive results for GAD autoantibodies appeared in 5 out of 72 patients (7.7%). Only one of them had a mutation for MODY, and all of these 5 patients were diagnosed with latent autoimmune diabetes of adults (LADA).

The gene mutations for MODY were assessed in the Marmara University genetics laboratory. In total, 67 (32 F/35 M; median age 36.1) diabetes patients were analyzed for 7 MODY mutations. They were categorized according to the pathogenicity identification as pathogenic MODY (+), variant of uncertain significance (VUS), and without any mutation (T2DM). Fifteen patients who had VUS mutations were excluded from the study for further evaluation. MODY (+) patients (n = 27) and T2DM patients (n = 25) were compared in terms of clinical and laboratory parameters.

### Genetic analysis

All patients were informed in person and their written consent was obtained. Genomic DNA was isolated from peripheral blood leucocytes using the QIAamp DNA Blood Mini QIAcube Kit (Qiagen, Hilden, Germany), according to the manufacturer’s protocols. All coding exons and exon-intron boundaries of seven genes that were associated with MODY ( *KCNJ11, ABCC8, INS, GCK, HNF4A, HNF1A, HNF1B* ) were amplified using the Multiplicom MODY MasterDx (Agilent, CA, USA) kit. Prepared library was sequenced on the Illumina Miseq platform (Illumina Inc., San Diego, CA, USA). The data were analyzed by the Sophia DDM data analysis software. In order to call variants, sequencing data was aligned to human reference genome, hg19. After amplifying targeted regions using designed primers, Sanger sequencing on ABI Prism 3500 Genetic Analyzer (Thermo Fisher Scientific, MA USA) was performed for the confirmation of the detected variants and segregation analysis. Novel variations were classified according to the American College of Medical Genetics and Genomics criteria ( 20 ). Mutation Taster, The Sorting Intolerant from Tolerant (SIFT) and deleterious annotation of genetic variants using neural networks (DANN) were used for computational pathogenicity prediction ( 21 ). The data of minor allele frequencies of variants were obtained from GnomAD ( 22 ).

### Statistical analysis

The distribution of the data was examined using the Shapiro-Wilk test. Normally distributed data between the two groups were compared with an independent samples *t* test, and non-normally distributed data between the two groups were compared with the Mann-Whitney U test. The difference between categorical variables was examined with Pearson’s *X^2^* test, and Fisher’s exact test. The descriptive statistics of the data are presented as mean and standard deviation, median (min-max), and n (%). All statistical analyses were conducted in the IBM SPSS Statistics 22.0 program with a significance level of 0.05.

## RESULTS

We evaluated 67 patients who were eligible for this study design. Twenty-seven of these patients (40.3%) were found to have pathogenic mutations grouped as MODY (+), and 15 were found to have VUS mutations (22.4%) and were excluded from the study. In MODY (+) subjects, mutations in *GCK* (MODY 2) were the most common followed by *HNF4A* (MODY 1). All pathogenic and likely pathogenic mutation types in the mutation-positive group were given in detail in Table 1 ( 23 - 33 ).

**Table 1:** Pathogenic and likely pathogenic variants

	Gene	Transcript	Variation	Variation Type	Status	dbSNP	ClinVar	Classification ACMG	HGMD	References
1	*GCK*	NM_000162	c.214G>A (p.Gly72Arg)	Missense	PR	rs193922289	P	P	CM023383	Lehto *et al* ., 1999 ( 23 )
2	*GCK*	NM_000162	c.506A>G (p.Lys169Arg)	Missense	PR	-	-	LP	CM141531	Flanagan *et al* ., 2014 ( 24 )
3	*GCK*	NM_000162	c.572G>A (p.Arg191Gln)	Missense	PR	rs886042610	LP/VUS	LP	CM012120	Massa *et al* ., 2001 ( 25 )
4	*GCK*	NM_000162	c.775G>A (p.Ala259Thr)	Missense	PR	rs1375656631	P	P	CM980894	Hattersley *et al* ., 1998 ( 26 )
5	*GCK*	NM_000162	c.898G>A (p.Glu300Lys)	Missense	PR	rs1255911887	-	LP	CM930305	Froguel *et al* ., 1993 ( 27 )
6	*GCK*	NM_000162	c.943C>T (p.Leu315Phe)	Missense	PR	-	-	LP	CM064013	Vits *et al* ., 2006 ( 28 )
7	*HNF1A*	NM_000545	c.392G>A (p.Arg131Gln)	Missense	PR	rs753998395	P	LP	CM961361	Yamagata *et al* ., 1996( 29 )
8	*HNF1B*	NM_000458	c.1390G>C (p.Gly464Arg)	Missense	Novel	-	-	LP	-	-
9	*HNF4A*	NM_001030003	c.110T>C (p.Leu37Pro)	Missense	Novel	-	-	LP	-	-
11	*HNF4A*	NM_001030003	c.1097C>G (p.Pro366Arg)	Missense	PR	rs193922469	LP	LP	-	-
12	*ABCC8*	NM_000352	c.1616A>G (p.Tyr539Cys)	Missense	PR	rs193922397	LP	LP	-	-
13	*ABCC8*	NM_000352	c.4055G>A (p.Arg1352His)	Missense	PR	rs28936370	P	P	CM042667	Magge *et al* ., 2004 ( 30 )
14	*ABCC8*	NM_000352	c.4306C>T (p.Arg1436*)	Nonsense	PR	rs193922402	P	P	CM112832	Powell *et al* ., 2011 ( 31 )
15	*ABCC8*	NM_000352	c.4631G>C (p.Ser1544Thr)	Missense	Novel	-	-	LP	-	-
16	*KCNJ11*	NM_000525	c.841C>T (p.Leu281Phe)	Missense	Novel	-	-	LP	-	-
17	*KCNJ11*	NM_000525	c.1019C>A (p.Pro340His)	Missense	PR	-	-	LP	CM144523	Mohnike *et al* ., 2014 ( 33 )
18	*KCNJ11*	NM_000525	c.1084G>A (Ala362Thr)	Missense	PR	rs755839409	-	LP	CM182438	Mohan *et al* ., 2018 ( 32 )

PR: previously reported; P: pathogenic; LP: likely pathogenic; VUS: variant of uncertain significance.

We detected four novel variations which were predicted to be likely pathogenic according to the ACMG guidelines ( 20 ). The novel likely pathogenic variants and pathogenicity evaluation details were presented in Table 2 . Related variations were segregated with the disease in three families. However, the patient carrying the *HNF4A* c.110T>C (p.Leu37Pro) variation declared that no family members were diagnosed with MODY, hence we were not able to screen the parents for MODY.

**Table 2:** Pathogenicity evaluation of novel variations

	Gene (Transcript ID)	Variation	Family History	Segregation	Mutation Taster	SIFT	DANN Score	GnomAD	Pathogenicity
1	*HNF1B* (NM_000458)	c.1390G>C (p.Gly464Arg)	+	Compatible	Disease Causing	Damaging	0.9986	0	LP
2	*HNF4A* (NM_001030003)	c.110T>C (p.Leu37Pro)	-	N/A	Disease Causing	Damaging	0.9964	0	LP
3	*ABCC8* (NM_000352)	c.4631G>C (p.Ser1544Thr)	+	Compatible	Disease Causing	Tolerated	0.9672	0	LP
4	*KCNJ11* (NM_000525)	c.841C>T (p.Leu281Phe)	+	Compatible	Disease Causing	Tolerated	0.9729	0	LP

In MODY (+) subjects, *GCK* mutations (MODY 2) (n = 12; 44.4%) were the most common followed by *HNF4A* mutations (MODY 1) (n = 3; 11.1%). When we compared MODY (+) (n = 27) and T2DM (n = 25) groups according to the laboratory parameters, no differences between the groups were found. C-peptides, HbA1c, lipid parameters, creatinine, glomerular filtration rate (GFR), microalbuminuria, vitamin D, and calcium were not statistically different between the groups ( Table 3 ).

**Table 3:** Comparison of laboratory parameters of the groups

	MODY (n:27)	T2DM (n:25)	p-value
C-peptide	1.60(0.01-3.18)	1.78(0.65-4.78)	0.145
FPG (mg/dL)	112(82-366)	109(74-329)	0.440
HbA1c (%)	6.70(5.8-10.5)	7(5.4-14.6)	0.962
LDL	101(38-201)	115(83-212)	0.068
HDL	49(26-65)	45(27-68)	0.283
Trig	91(32-558)	120(37-381)	0.180
U acid	4.70(2.80-6.40)	4.80(1.80-7.56)	0.705
Crea	0.61(0.21-0.96)	0.69(0.36-2.63)	0.118
GFR	143.79(55.56-315)	114.26(62.03-1725)	0.213
Microalbuminuria	43(32-52)	43(37-52)	0.842
Ca	9.40(8.3-10.3)	9.50(8.8-10.8)	0.355
P	3.20(2-5)	3.40(2-4.6)	0.573
PTH	39.13(18.90-83.27)	38.53(10.11-58.13)	0.848
Vitamin D	16.82(7.03-43)	15.54(4.72-48.77)	0.952

Regarding demographic parameters and diabetes history, median age was 36.1 years, and there appeared to be no difference between the groups. BMI and gender were similar between the groups ( Table 4 ). Diabetes diagnosis age was younger in the MODY (+) group, but the difference was not statistically significant. Sixty-six percent of MODY (+) subjects had diabetes history for 3 consecutive generations in their family, compared to 32% of T2DM patients, which was statistically significant ( *p* = 0.008). The groups were similar in terms of diabetes complications. Pharmacologic treatment modalities were not very different between the groups, but we found that MODY (+) patients tended to start insulin therapy at a younger age than the negative group-did; thus, their insulin duration time was longer than that of the negative group, although their insulin doses were lower than the T2DM group. We evaluated all patients according to concomitant comorbidities such as hypertension, hyperlipidemia, and hepatosteatosis. More patients in the T2DM group had hypertension (40%) than in the MODY (+) group (7.7%), which was statistically significant ( *p* = 0.007). Other comorbidities were similar between the groups ( Table 4 ).

**Table 4:** Comparison of the groups according to demographic results and history of diabetes

		MODY (n:27)	T2DM (n:25)	p-value
Gender (F/M)				
	Female	11 (40.7%)	14 (56%)	0.271
	Male	16 (59.3%)	11 (44%)	
Age (years)		34.22 ± 16.26	40.96 ± 11.77	0.092
BMI (kg/m2)		28.64 (20.3-44.1)	27.63 (20.36-52.49)	0.624
DM diagnosis age		28.11 ± 16.41	33.40 ± 11.69	0.185
3 generation DM		18 (66.6%)	8 (32%)	**0.008**
Retinopathy (n)		0	3 (12%)	1
Nephropathy (n)		4 (15.4%)	6 (24%)	0.499
Neuropathy (n)		2 (7.7%)	4 (16%)	0.419
OAD		1(1-3)	1(1-3)	0.725
OAD duration (years)		6.50(1-23)	3(1-20)	0.138
Insulin starting age (years)		21.82 ± 10.74	32.43 ± 10.54	**0.021**
Insulin duration (years)		11(2-23)	5(1-16)	0.231
<0.5 IU/kg insulin requirement (n)		17 (73.9%)	16 (69.6%)	0.743
Hypertension (n)		2 (7.7%)	10 (40%)	**0.007**
Hepatosteatosis (n)		9 (34.6%)	8 (32%)	0.843
Hyperlipidemia (n)		3 (11.5%)	7 (28%)	0.173

## DISCUSSION

In this study, we analyzed the accuracy and parallelism of our mutation-requesting criteria with the presence of mutations among adult diabetic patients, and also compared the clinical and laboratory results of mutation (+) and (-) groups. We found MODY mutation positivity in 40.3% of all study subjects and the most common mutation was *GCK* (44.4%) in our study population. The frequency of MODY in T2DM patients varies by country, with 21% in the USA, 27% in the UK, 31% in Norway, 39% in the Netherlands, 19% in Japan, and to our knowledge, 29% in the Turkish pediatric population ( 17 , 34 ). MODY subtype frequencies vary by region. Haliloglu and cols. ( 17 ) showed that the most common MODY mutation is *GCK* in pediatric diabetic patients in Turkey (almost 25%), whereas in the SEARCH study *HNF1A* mutation was found to be the most common MODY subtype among the young diabetic population in the USA (10). Almost all studies about MODY were performed in pediatric groups, and the most common MODY subtype was usually the *GCK* mutation in European countries, higher than in Asian countries such as Japan and Korea ( 35 , 36 ). In our study, pathologic *GCK* mutations were the most common, followed by *HNF4A* mutations in the adult MODY (+) group; similar to what was found in the pediatric population in Turkey. These results are consistent with the distribution in other Southern European populations ( 15 , 34 , 37 ).

Genetic testing is the gold standard for diagnosing MODY and it can be utilized for planning a treatment strategy according to the mutation type. However, genetic testing is often expensive and widely accessible. Thus, careful consideration is required when determining which patients need to undergo genetic testing. According to the results of some studies, most of which were conducted in childhood diabetes patients, some criteria were established, and genetic analysis was recommended for patients who met these criteria. Shields and cols. ( 3 ) developed a prediction model to determine the probability of MODY in patients with young-onset diabetes. They predicted that positive C-peptides and negative autoantibodies were strongly suggestive of MODY compared to T1DM. On the other hand, they claimed that the presence of insulin resistance and high BMI could be clinical markers for T2DM.

MODY is a genetically heterogeneous disease and to date 14 genes ( *GCK, HNF1A* , *HNF4A, HNF1B, INS, KCNJ11, ABCC8, PDX1, NEUROD1, KLF11, CEL, PAX4, BLK* and *APPL1* ) were associated with MODY. However, *GCK* and *HNF1A* mutations are detected in approximately half of the MODY patients ( 25 ). We detected four novel variations which were predicted to be likely pathogenic according to the ACMG guidelines. None of these four novel variations were reported in population studies (GnomAD) and they were not present in 200 Turkish control chromosomes. The novel likely pathogenic variants were analyzed using in silico analysis tools stated in Table 2 . Family members were screened for the detected variations and it was shown that related variations co-segregated with the disease in three families. However, the patient carrying the *HNF4A* c.110T>C (p.Leu37Pro) variation declared that no family members were diagnosed as MODY and we were not able to screen the parents for MODY. In total, 180 variations were reported in The Human Gene Mutation Database Professional in *HNF4A* gene of which most are missense variations. There are several studies reporting molecular findings in MODY cases in Turkish or other populations. However, it is still possible to encounter novel variations due to high allelic heterogeneity in MODY ( 26 , 27 ).

Few studies have compared adult T2DM patients with MODY patients in terms of clinical and laboratory parameters. Chambers and cols. ( 38 ) compared 75 MODY (-) and 22 MODY (+) patients. The positive group had a lower HbA1c, and their family history of diabetes was significantly longer; most of them had not undergone any pharmacological treatment. According to these findings, they reported that MODY could be suspected in youth diabetes patients with negative antibodies and preserved C-peptides. Zhang and cols. ( 11 ) reported that MODY(+) Chinese patients were younger at diagnosis, and had a longer duration of diabetes, higher fasting plasma glucose, lower C-peptides, lower BMI, lower HOMA and lower triglycerides compared with early-onset T2DM patients. A recent study on 263 Japanese patients ( 35 ) showed that mutation-positive patients had a lower BMI and insulin resistance compared to mutation-negative diabetics; and were also younger at the time of the diagnosis. Based on previous studies, Jang ( 39 ) suggests MODY genetic analysis in adult diabetic patients if they were diagnosed before the age of 30, if β-cell antibodies are negative, and if they have a family history of diabetes and BMI ≤ 30 kg/m^2^ without insulin resistance.

In our study, family history was the most significant distinguishing feature among the clinical MODY diagnosis criteria. MODY (+) subjects had a significantly long diabetes history of 3 consecutive generations in their family (66%) compared to T2DM patients (32%) ( *p* = 0.008). Diabetes diagnosis age was younger in the MODY (+) group compared to the mutation-negative group, but this difference was not statistically significant ( *p* = 0.092). Since most patients with the *GCK* mutation can continue without treatment for many years, the age of diagnosis may be advanced. In our study, we have found that the age of diagnosis of the MODY (+) group was higher than expected. This might be caused by the presence of *GCK* mutation in the majority of the patients and the fact that some of these patients were incidentally diagnosed by genetic analysis when their first degree relatives were diagnosed with mutation-positive T2DM. Contrary to other studies, we found no significant differences in C-peptides, BMI, or HbA1c between the groups. Similarly, in another study from Korea ( 36 ) comparing 23 mutation-positive patients with 17 early-onset T2DM patients in terms of clinical and metabolic profiles, no differences were found for age at diagnosis, BMI, C-peptides, and fasting and postprandial glucose levels. Interestingly, we found that mutation-positive patients start insulin therapy at younger ages than the T2DM patients. On the other hand, we had expected that *GCK* mutation-positive patients would not need any treatment for a long time. This could be because other mutations may cause uncontrolled aggressive hyperglycemia. In our study population, hypertension was the unique comorbidity accompanying to diabetes in mutation-negative patients. Although microvascular complication frequency was quite higher in T2DM patients, there were no statistically differences between the groups according to diabetes complications, duration of diabetes, or insulin usage.

All these study results show that previously suggested diagnostic criteria for MODY ( 10 , 40 , 41 ) might not be sufficient to predict MODY patients. According to current studies, C-peptide level seems to be more of an important criterion for T1DM. No difference was found in our patient group in terms of HbA1c and BMI; thus, they might not be sufficient criteria to predict MODY alone. According to the present study results, MODY mutation positivity is most probable in young autoantibody negative diabetes patients diagnosed before 30 years of age; who have a first-degree family history of diabetes. Screening for MODY would be an appropriate approach in young patients who typically do not fit the T2DM profile and have a first-degree family history of diabetes.

One limitation of our study was the small number of patients because we only recruited patients who were suspected of MODY and who were referred for genetic analysis. Therefore, we did not define specific inclusion criteria for the patients. Another limitation of our study was that all existing mutations could not be examined. We screened for the single nucleotide variations (SNVs) and small deletions or insertions in only 7 of these genes’ coding regions. We were not able to exclude the copy number variations (CNVs), variations in noncoding regions of these genes and the rest of the MODY related genes in MODY (-) group.

In conclusion, according to the results of this study, it would be meaningful to investigate MODY mutations in T2DM patients who were diagnosed before the age of 30, who have a family history of diabetes in their first-degree relatives, and who are autoantibody negative. Other parameters are not very valuable for screening MODY mutation in every young diabetic patient.
